# Enhanced response to pulmonary *Streptococcus pneumoniae* infection is associated with primary ciliary dyskinesia in mice lacking Pcdp1 and Spef2

**DOI:** 10.1186/2046-2530-2-18

**Published:** 2013-12-20

**Authors:** Casey W McKenzie, Joshua M Klonoski, Taylor Maier, Glenda Trujillo, Peter F Vitiello, Victor C Huber, Lance Lee

**Affiliations:** 1 Sanford Children’s Health Research Center, Sanford Research/USD, 2301 E. 60th St. N, Sioux Falls, SD 57104, USA; 2 Division of Basic Biomedical Sciences, Sanford School of Medicine of the University of South Dakota, 414 E. Clark St, Vermillion 57069 SD, 57069, USA; 3 Department of Pathology, Stony Brook University School of Medicine, Stony Brook, NY 11794, USA; 4 Department of Pediatrics, Sanford School of Medicine of the University of South Dakota, Sioux Falls, SD 57105, USA

**Keywords:** Cilia, Lung, Primary ciliary dyskinesia, *Streptococcus pneumoniae*

## Abstract

**Background:**

Lower airway abnormalities are common in patients with primary ciliary dyskinesia (PCD), a pediatric syndrome that results from structural or functional defects in motile cilia. Patients can suffer from recurrent bacterial infection in the lung, bronchiectasis, and respiratory distress in addition to chronic sinusitis, otitis media, infertility, and laterality defects. However, surprisingly little is known about the pulmonary phenotype of mouse models of this disorder.

**Results:**

The pulmonary phenotype of two mouse models of PCD, *nm1054* and *bgh*, which lack Pcdp1 and Spef2, respectively, was investigated by histological and immunohistochemical analysis. In addition, both models were challenged with *Streptococcus pneumoniae*, a common respiratory pathogen found in the lungs of PCD patients. Histopathological analyses reveal no detectable cellular, developmental, or inflammatory abnormalities in the lower airway of either PCD model. However, exposure to *S. pneumoniae* results in a markedly enhanced inflammatory response in both models. Based on analysis of inflammatory cells in bronchoalveolar lavage fluid and flow cytometric analysis of cytokines in the lung, the *bgh* model shows a particularly dramatic lymphocytic response by 3 days post-infection compared to the *nm1054* model or wild type animals.

**Conclusions:**

Defects in ciliary motility result in a severe response to pulmonary infection. The PCD models *nm1054* and *bgh* are distinct and clinically relevant models for future studies investigating the role of mucociliary clearance in host defense.

## Background

*Streptococcus pneumoniae*, an anaerobic, gram-positive bacterium, is the primary cause of bacterial pneumonia and a major cause of sinusitis, otitis media, and bacterial meningitis [1-3]. Symptoms of pneumococcal pneumonia commonly include a persistent cough, excess production of sputum, shortness of breath, and fever, with most patients also showing evidence of a pulmonary infiltrate [1,3]. Despite the effectiveness of antibiotics and immunizations, pneumococcal pneumonia continues to result in respiratory failure, hospitalization, and mortality in many patients, particularly young children, the elderly, and individuals with additional underlying medical conditions [1,2,4]. It is estimated that over one million childhood deaths are associated with pneumococcal pneumonia each year [3].

The response to respiratory pathogens involves mucus secretion, clearance by ciliary motility, and an inflammatory response [5-7]. Because of the role of cilia in this response, pulmonary disease is a major component of primary ciliary dyskinesia (PCD), an autosomal recessive, pediatric syndrome that results from defects in ciliary and flagellar motility and is commonly characterized by chronic upper and lower respiratory infection, otitis media, male infertility, and situs inversus, as well as less frequent associations of hydrocephalus and female infertility [8-13]. Because mucociliary clearance is critical for lung maintenance and host defense, PCD patients typically have a variety of pulmonary defects as early as the neonatal period [5,14-18]. Several large or longitudinal clinical studies analyzing pulmonary function and disease in diagnosed PCD patients found a variety of common conditions [19-33]. Patients typically suffered from recurrent pneumonia, a chronic cough, excess sputum production, and severe bronchiectasis. Neonatal respiratory distress was frequently reported in infants, while airway obstruction, atelectases, and a progressive, irreversible decline in pulmonary function were commonly seen in older patients. Bacteria were identified in the sputum from most patients, with *Haemophilus influenzae*, *Streptococcus pneumoniae*, and *Staphylococcus aureus* being the most common. *Pseudomonas aeruginosa*, a pathogen commonly found in the lungs of cystic fibrosis patients, was only rarely identified in PCD patients.

Despite the strong correlation of lower airway disease with PCD in patients, there is a surprising paucity of information about lung disease in mouse models of PCD or the usefulness of those models for developing improved therapies for lung disease. Although several mouse models with PCD or PCD-related phenotypes have been described [11,12], pulmonary defects have only been reported in three. Studies involving lower airway disease are complicated in some models that exhibit early lethality due to hydrocephalus or laterality defects, but several other models have been described on genetic backgrounds that are less susceptible to severe hydrocephalus and therefore live a longer lifespan [12]. Mice lacking both sperm associated antigens 6 (Spag6) and 16 L (Spag16L), which have been shown to interact in the cilium, have severe lower airway disease characterized by pneumonia, atelectases, and hemorrhage [34,35]. Despite the presence of other PCD phenotypes, respiratory abnormalities were not reported in mutants lacking either individual protein [36,37], possibly due to the functional relationship between the two proteins. Loss of Spag17 also results in PCD, with homozygous mutants exhibiting an accumulation of fluid in their lungs, damage to the alveolar cells, and a failure to thrive [38]. Severe lower airway disease was also observed in mice lacking the Wnt inhibitor Chibby and is characterized by abnormal lung pathology, reduced proliferation and differentiation of respiratory epithelial cells, and an inability to clear *P. aeruginosa* after infection [39,40]. While these studies indicate that mouse models may be valuable tools for better understanding and treating the array of pulmonary phenotypes associated with PCD, they are limited in number and have not yet explored the response to the respiratory pathogens most commonly observed in human PCD patients.

PCD has been previously reported in the *nm1054* and *big giant head* (*bgh*) mouse models and is characterized, in both cases, by hydrocephalus, male infertility, and upper respiratory abnormalities [41,42]. The phenotypes in the *nm1054* and *bgh* models are caused by loss of ciliary proteins primary ciliary dyskinesia protein 1 (Pcdp1) and sperm flagellar protein 2 (Spef2), respectively [41,42]. The PCD phenotypes demonstrate that both genes are required for proper ciliary function, although mutations have not yet been identified in human PCD patients. In the *nm1054* mutant, there is a marked accumulation of mucus in the upper airway [41], while the *bgh* mutant has a similar accumulation of mucus accompanied by an infiltration of neutrophils [42], which is indicative of an inflammatory response in the airway [17,43]. The respiratory epithelial cilia have a normal ultrastructure in both models [41,42]. However, the ciliary beat frequency (CBF) is decreased by approximately 25% in the *nm1054* mouse and approximately 17% in the *bgh* mouse [41,42], likely resulting in the inability to clear mucus in the airway. Given these findings, it is expected that a defect in mucociliary clearance would also affect the lower airway and predispose these models to severe respiratory infection.

In this study, we conducted a comprehensive histopathological analysis of the lungs of both the *nm1054* and the *bgh* model. Despite the pathological findings in the upper airway and the pulmonary abnormalities observed in other PCD models, there are no major cellular, developmental, or inflammatory abnormalities in the lower airway of either the *nm1054* or the *bgh* mutant. In contrast, there is a markedly enhanced acute inflammatory response in both models after infection with *S. pneumoniae*, with the *bgh* model showing a particularly dramatic lymphocytic response within 3 days of infection. These findings demonstrate that defects in ciliary motility in both PCD mutants result in severe pulmonary infection and establish clinically relevant models for future studies involving the role of mucociliary clearance in host defense.

## Methods

### Mice

The *nm1054*[41] and *bgh*[42] lines were maintained on both the C57BL/6 J (B6) and 129S6/SvEvTac (129) backgrounds as previously described. All phenotypic analyses were performed on (B6x129)F1 animals at least 8 weeks old. All procedures were approved by either the Sanford Research/USD or the University of South Dakota Institutional Animal Care and Use Committee.

### Histology

Three wild type, three *nm1054*, and three *bgh* lungs were inflated with 10% phosphate-buffered formalin using an 18-gauge catheter inserted into the trachea. The lungs were removed from the body cavity and immersion fixed in 10% phosphate-buffered formalin. The fixed tissue was embedded in paraffin wax on a Leica 300 ASP tissue processor, serially sectioned through the airway, and stained with hematoxylin and eosin on a Sakura Tissue-Tek automated stainer. Sections were analyzed qualitatively by light microscopy on an Olympus IX71 microscope.

### Immunohistochemistry

Sections from the three wild type, three *nm1054*, and three *bgh* lungs used for histology were stained with primary and secondary antibodies using the BenchMark XT automated slide staining system (Ventana Medical Systems, Inc.). The mouse acetylated tubulin antibody (Sigma Aldrich) was used at 1:6,000 and incubated for 30 minutes at 37°C. The goat Clara cell secretory protein (CCSP) (courtesy of Barry Stripp, Duke University Medical Center, Durham, NC, USA) and rabbit prosurfactant protein B (Chemicon) antibodies were used at 1:250 and incubated for 1 hour at 37°C. The Syrian hamster T1α glycoprotein 38 kD mucin-like antibody (Hybridoma Bank) was used at 1:1,000 and incubated for 1 hour at 37°C. All secondary antibodies were from Jackson ImmunoResearch. Biotin SP-conjugated AffiniPure goat anti-rabbit and goat anti-Syrian hamster secondary antibodies were used at 1:1,000, and the Biotin SP-conjugated AffiniPure donkey anti-mouse and donkey anti-goat secondary antibodies were used at 1:500. Staining was detected using the Ventana iView DAB kit, and the slides were counterstained with hematoxylin and qualitatively analyzed by light microscopy on an Olympus IX71 microscope.

### Alveolar chord length measurement

Images of lung sections from the three wild type, three *nm1054*, and three *bgh* mice analyzed by histology above were taken on a Nikon 90i microscope using a 10X objective and at a 1,600 × 1,200 resolution. Alveolar cord length was measured as previously described using the Image J software [44,45], and statistical significance was determined using the Student’s *t*-test.

### Bacterial infection

Mice anesthetized with isoflurane were infected with the TIGR4 strain of *S. pneumoniae* (courtesy of Carlos Orihuela, University of Texas Health Science Center, San Antonio, TX, USA) by nasal instillation with a total of 5 × 10^4^ CFU in 10 μL inoculated into the left nostril. Body weights were collected for all mice prior to inoculation and at the time of death. Nasal carriage was assessed in surviving mice prior to euthanasia at 1, 2, or 3 days post-infection by instilling 25 μL of phosphate buffered saline into the left nostril, recovering, serially diluting, and plating on 10% sheep red blood cell agar [46]. Lung titer was assessed following euthanasia at 1, 2, or 3 days post-infection by homogenizing the left lung with an IKA T10 Basic S1 disperser, serially diluting the homogenate, and plating on 10% sheep red blood cell agar as previously described. Statistical significance was determined using the Student’s *t*-test. The following numbers of mice were analyzed for nasal carriage and lung titer: on day 1, four wild type, four *nm1054*, and four *bgh*; at day 2, four wild type, four *nm1054*, and eight *bgh*; and at day 3, four wild type, four *nm1054*, and eight *bgh*.

### Bronchoalveolar lavage

Bronchoalveolar lavage fluid was isolated from four uninfected wild type, four uninfected *nm1054*, and three uninfected *bgh* mice, as well as three wild type, three *nm1054*, and three *bgh* mice 2 and 3 days after infection with *S. pneumoniae*. Lungs were inflated with 1 mL of Hank’s Balanced Salt Solution (HBSS) media containing 5 mM EDTA using the inflation procedure described above, and the fluid was collected through the same catheter using a separate syringe. Inflation and collection was repeated six times. Lavage fluid was centrifuged at 400 × *g* for ten minutes at 4°C. The supernatant was removed, and the cells were pooled and resuspended in 200 μL of HBSS media containing 5 mM EDTA. The cells were loaded onto slides and spun in a StatSpin Cytofuge 2 cytocentrifuge (Iris Sample Processing, Inc.) at 600 rpm for 6 minutes. The slides were fixed with methanol, stained with the Camco differential stain kit, and visualized on an Olympus IX71 microscope.

### Cytokine profiling

Cytokines were analyzed in lungs from uninfected mice (day 0) and 1, 2, or 3 days after infection with *S. pneumoniae*. Right lungs were homogenized as described above and centrifuged at 5,000 rpm for 1 minute. Cytokines in the supernatant were analyzed using the Cytometric Bead Array Mouse Th1/Th2/Th17 Cytokine Kit (BD Biosciences) and an Accuri C6 flow cytometer (BD Biosciences) following manufacturer’s instructions. Cytokine levels were normalized to internal standards, and a standard curve was generated using the GraphPad Prism 4 software. Statistical significance was determined using the Student’s *t*-test. The following numbers of mice were analyzed for cytokines: on day 0, four wild type, four *nm1054*, and four *bgh*; on day 1, four wild type, four *nm1054*, and four *bgh*; on day 2, four wild type, four *nm1054*, and eight *bgh*; and on day 3, four wild type, four *nm1054*, and eight *bgh*.

## Results

### Absence of lower airway pathology in *nm1054* and *bgh* lungs

**Figure 1:**
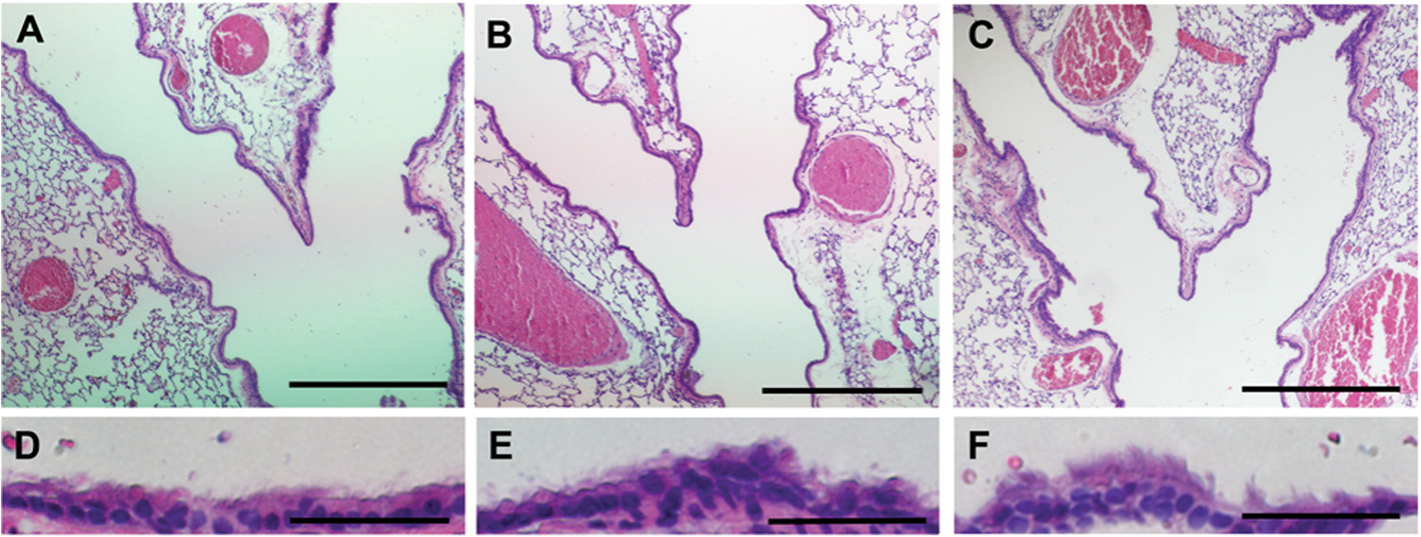
**Histopathology of mutant lungs.** Sections of wild type **(A,D)**, *nm1054***(B,E)**, and *bgh***(C,F)** lungs through the major airway showing an absence of major defects in either mutant. High magnification images **(D–F)** show that cilia are present on the epithelial surface of both mutants. Sections are stained with hematoxylin and eosin. Scale bars represent either 500 **(A–C)** or 50 **(D–F)** μm.

**Figure 2:**
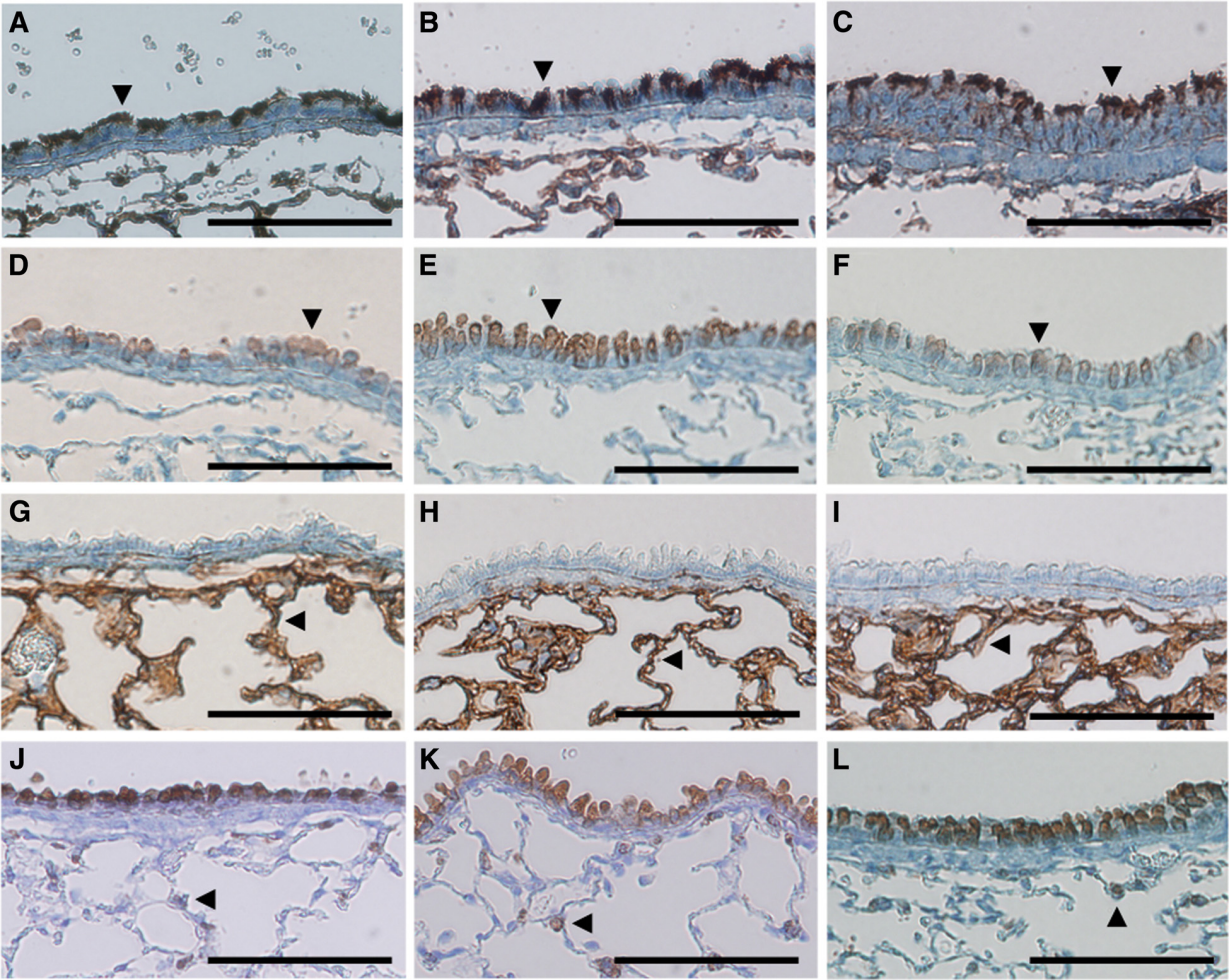
**Absence of cellular defects in mutant lungs.** Immunohistochemical analysis of cell-specific markers in wild type **(A,D,G,J)**, *nm1054***(B,E,H,K)**, and *bgh***(C,F,I,L)** lungs. **(A–C)** Acetylated tubulin, a marker of epithelial cilia showing a normal appearance and distribution of cilia in both mutants. **(D–F)** CCSP, a marker of Clara cells. **(G–I)** T1α, a marker of alveolar type I cells. **(J–L)** Prosurfactant protein B, a marker of alveolar type II cells and Clara cells. Arrowheads indicate the stained epithelial **(A–C)**, Clara **(D–F)**, alveolar type I **(G–I)**, and alveolar type II **(J–L)** cells. Scale bars represent 100 μm.

Because pulmonary abnormalities are common in patients with PCD, we investigated the lung phenotype in both the *nm1054* and *bgh* mutants. Histological analysis of lung architecture revealed no significant abnormalities or any major damage to tissue structure in either model (Figure 1A–C). Higher magnification images of the epithelia lining the airway demonstrate that the epithelial cilia are intact in both mutants (Figure 1D–F). To confirm these histological findings, a more detailed analysis of tissue architecture was enabled by immunohistochemical analysis of markers specific to airway cell types. Acetylated tubulin, which is expressed in the ciliated epithelial cells lining the airway, is properly expressed and localized to the cilia in both mutants (Figure 2A–C), confirming the normal appearance of respiratory epithelial cilia. Expression of CCSP, a marker of the Clara cells that also line the airway, shows a normal appearance and distribution of this epithelial cell type (Figure 2D–F). The alveolar epithelial cells were detected with antibodies to T1α and Prosurfactant protein B, which are markers for the type I and type II alveolar cells, respectively. Proper expression was detected for both T1α (Figure 2G–I) and Prosurfactant protein B (Figure 2J–L) in both PCD mutants, indicating that there are no significant abnormalities in the alveolar cell types. In addition, measurements of alveolar chord length showed no significant differences in the alveolar airspace in either the *nm1054* or the *bgh* lung (Additional file 1). Prosurfactant protein B is also expressed in the Clara cells, and normal expression in both mutants confirms the presence and normal distribution of this cell type. Proper expression of these cell-specific markers indicates that there are no developmental defects or secondary damage to the airway surface due to defective mucociliary clearance.

**Figure 3:**
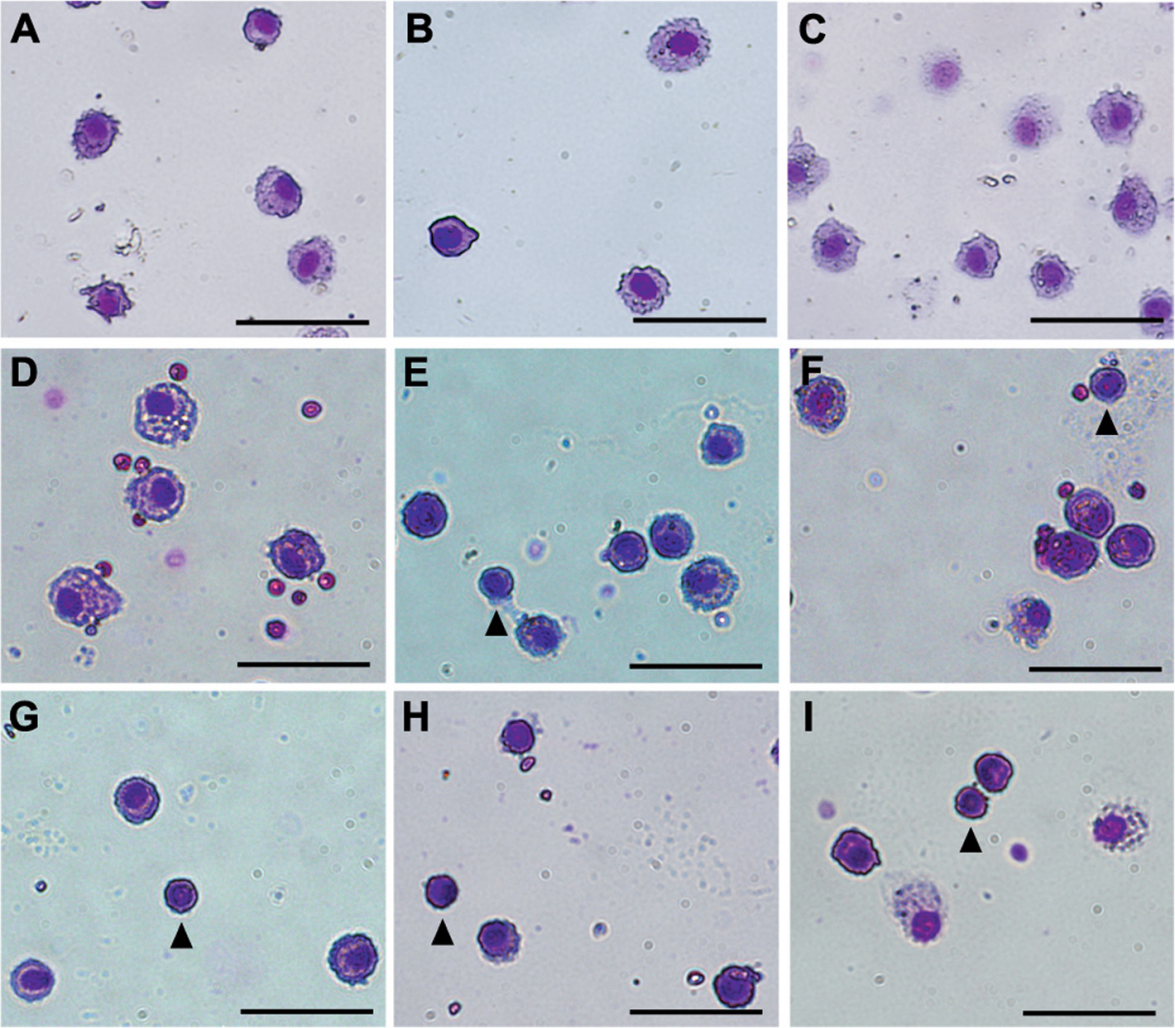
**Inflammatory cells in mutant lungs.** Representative images of inflammatory cells in BAL fluid from wild type **(A,D,G)**, *nm1054***(B,E,H)**, and *bgh***(C,F,I)** lungs. BAL fluid was analyzed in uninfected animals **(A–C)** and either 2 days **(D–F)** or 3 days **(G–I)** after infection with *S. pneumoniae*. Examples of lymphocytes in infected lungs are indicated by arrowheads. Cells were stained with the Camco Stain Pak differential staining kit. Scale bars represent 50 μm.

Because previous histological analysis of the maxillary sinus cavity of *bgh* mutants indicated the presence of an inflammatory response [42], we investigated whether an inflammatory response was active in the lower airway of both models. Presence or absence of inflammatory cells in the lower airway of *nm1054* and *bgh* mutants was assessed in bronchoalveolar lavage (BAL) fluid. The inflammatory cells in wild type lungs consist primarily of resident and circulatory macrophages (Figure 3A; Table 1). This is indicative of a healthy respiratory system, where macrophages typically comprise 95% of inflammatory cells [43]. Consistent with an absence of abnormal pathology in the mutant lungs, only resident alveolar macrophages were detected in BAL fluid from *nm1054* (Figure 3B; Table 1) and *bgh* (Figure 3C; Table 1) animals. There was no evidence of polymorphonuclear leukocytes or lymphocytes in the airway of either mutant. These findings suggest that, despite evidence of mucus accumulation and an inflammatory response in the upper airway, a defect in respiratory epithelial ciliary motility does not result in a detectable phenotype in the lungs of either the *nm1054* or the *bgh* mutant.

**Table 1:** Inflammatory cells in BAL fluid

**Mouse line**	**Day post-infection**	**Total number of cells counted**	**Resident macrophages (%)**	**Infiltrating macrophages (%)**	**T lymphocytes (%)**
Wild type	Uninfected	272	42	56	-
Wild type	2	441	79	12	6
Wild type	3	537	5	78	16
*nm1054*	Uninfected	184	99	-	-
*nm1054*	2	370	19	56	24
*nm1054*	3	513	-	53	42
*bgh*	Uninfected	261	99	-	-
*bgh*	2	632	56	12	26
*bgh*	3	463	-	17	82

Cell types were counted in pooled representative fields from three mice in each group in a blinded manner, and approximate percentages of the total number are shown. Percentages less than five are indicated by a dash, and only cell types representing at least 5% of the total cells are included.

### Response to *Streptococcus pneumoniae* infection in *nm1054* and *bgh* mutants

The surprising absence of a pulmonary phenotype in *nm1054* and *bgh* mutants could be due to a milder defect in mucociliary clearance in the lower airway. It is also possible that the lower airway of these models may be impervious to any significant damage in a clean environment without a respiratory challenge. To investigate whether *nm1054* and *bgh* mutants might exhibit a lower airway phenotype in response to a challenge, we infected both models with *S. pneumoniae*, a common respiratory pathogen seen in the airway of human PCD patients. Infection appeared to result in increased morbidity in both mutants, with one *nm1054* and one *bgh* mouse dying approximately 3 days after infection. Body weights for infected wild type, *nm1054*, and *bgh* mice remained largely unchanged for mice euthanized for analysis of inflammation up to 3 days post-infection (Additional file 2). However, those mice that died as a result of infection exhibited weight loss and wasting prior to death, with the *nm1054* mouse weighing approximately 76% of its initial weight prior to infection and the *bgh* mouse weighing approximately 88% of its initial weight, suggesting a substantial decline in health after infection. In contrast, none of the infected wild type controls exhibited any sign of abnormal health or expired prior to euthanasia.

**Figure 4:**
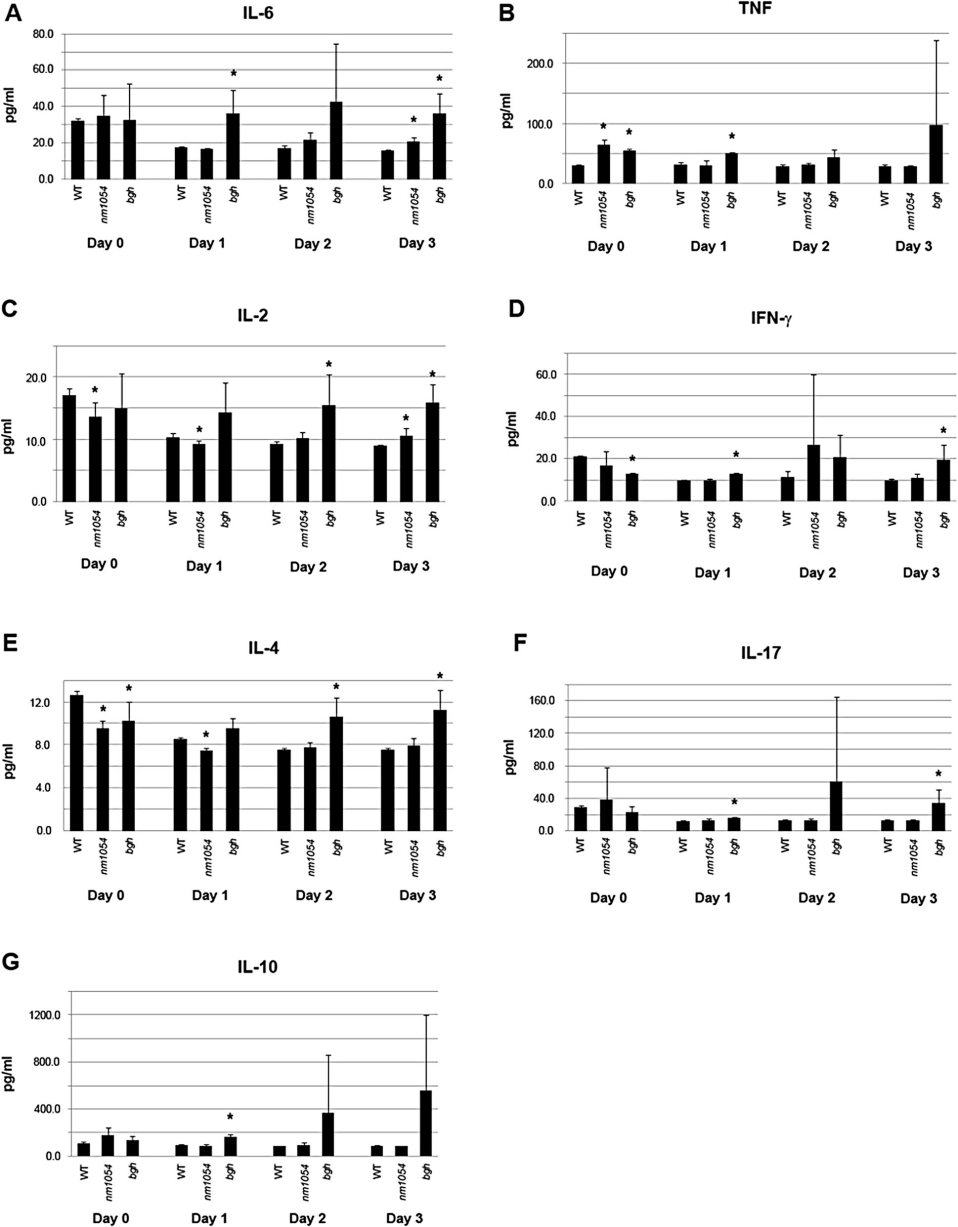
**Enhanced cytokine levels in response to *S. pneumoniae *** **infection.** Levels of IL-6 **(A)**, TNF **(B)**, IL-2 **(C)**, IFNγ **(D)**, IL-4 **(E)**, IL-17 **(F)**, and IL-10 **(G)** were measured in wild type, *nm1054*, and *bgh* lung homogenates by flow cytometry using a cytometric bead array mouse Th1/Th2/Th17 cytokine kit. Mice were infected with *S. pneumoniae* on day 0, and cytokine levels were measured on days 0, 1, 2, and 3. Cytokine levels are normalized to internal standards. Statistical significance relative to wild type (*P* <0.05) is indicated by an asterisk.

To investigate the effect of ciliary dysfunction, wild type, *nm1054*, and *bgh* mice were analyzed for an acute inflammatory response during the 3 days after infection. During this time, there were no significant changes in bacterial nasal carriage or lung titer (Additional file 3), suggesting an inability to clear the pathogen within this period. Analysis of cells in BAL fluid on days 2 and 3 after infection demonstrated a clear immune response in wild type, *nm1054*, and *bgh* mice. Infected wild type mice exhibited a shift from activated resident macrophages on day 2 to activated infiltrating macrophages differentiated from circulatory monocytes on day 3, when the activated infiltrating macrophage population comprised approximately 78% of the cells in the BAL fluid (Figure 4D,G; Table 1). In contrast to the wild type response, the *nm1054* BAL fluid shows a substantial increase in the number of activated macrophages and T lymphocytes on day 2 (Figure 4E; Table 1). By day 3, there is almost an equivalent presence of activated macrophages (53%) and T lymphocytes (42%) (Figure 4H; Table 1), demonstrating an enhanced lymphocytic response to *S. pneumoniae* infection relative to wild type. The most dramatic response to bacterial challenge was observed in *bgh* lungs. As in the *nm1054* mutant, day 2 shows a shift to activated macrophage and infiltrating T lymphocyte populations (Figure 4F; Table 1). By day 3, however, T lymphocytes comprise approximately 82% of the inflammatory cell population (Figure 4I; Table 1). This strong lymphocytic presence demonstrates a particularly aggressive immune response in *bgh* lungs compared to wild type or *nm1054* mice. Several other cell types, including neutrophils, eosinophils, and dendritic cells, were observed in infected lungs at low frequency, but these numbers were negligible compared to the infiltration of macrophages and T lymphocytes.

The acute inflammatory response observed in BAL fluid is supported by flow cytometric analyses of cytokines in lung lysates, which were performed in uninfected lungs and at days 1, 2, and 3 after infection with *S. pneumoniae* (Figure 4). The cytokine profiles show a marked response to infection in the *bgh* mutant. Early infection cytokines interleukin 6 (IL-6) and tumor necrosis factor (TNF), which are both secreted by macrophages, showed a statistically significant increase (*P* <0.05 by Student’s *t*-test) in the *bgh* lung as early as day 1 (Figure 4A,B), indicating the onset of an inflammatory response. Both cytokines remained at either significantly higher or elevated levels through day 3 relative to wild type. Lack of statistical significance is likely due to typical variation in immune response within the mouse sampling. In addition, there is either an upward trend or a significant increase in the level of IL-2, interferon gamma (IFNγ), IL-4, and IL-17, all of which are indicators of T lymphocyte activation, by days 2 and 3 (Figure 4C-F). The elevated levels of lymphocytic cytokines are consistent with the aggressive infiltration of lymphocytes observed in *bgh* BAL fluid (Figure 3, Table 1). Finally, an increase in the level of anti-inflammatory cytokine IL-10, which is produced by activated T regulatory cells, is observed on days 1 through 3 (Figure 4G). In contrast to the cytokine profile of *bgh* lungs, a less striking response is observed in *nm1054* lungs. By day 3, a significant increase was only observed for IL-6 (Figure 4A), which is indicative of an early macrophagic response, and IL-2 (Figure 4C), which is consistent with the mild lymphocytic response observed in the *nm1054* BAL fluid (Figure 3, Table 1).

## Discussion

Although chronic lung disease is commonly associated with PCD in human patients, there are no significant histopathological defects in the lungs of mice lacking either ciliary protein Pcdp1 or Spef2, despite the presence of upper airway abnormalities, male infertility, hydrocephalus, and a decrease in ciliary motility [41,42]. However, infection with *S. pneumoniae* results in an enhanced acute inflammatory response in both PCD models. It is likely that the defects in ciliary motility result in perturbed mucociliary clearance of the pathogen, which leads to the enhanced immune response. Persistent bacterial infection is indicated by unchanged levels in nasal carriage and lung titer over 3 days after inoculation with *S. pneumoniae*.

The absence of a detectable phenotype in uninfected mutant lungs may in part be due to the housing of these animals in a clean, pathogen-free environment void of any pulmonary challenges. In this environment, the inflammation previously observed in the upper airway of the *bgh* mutant may be sufficient to protect the lower airway from any significant pathology [42]. Alternatively, absence of an unchallenged pulmonary phenotype could result from a milder defect in mucociliary clearance in the lower airway that is related to the functions of Pcdp1 and Spef2, which is supported by the presence of lung disease in unchallenged mice lacking other ciliary proteins, such as Spag17 or both Spag6 and Spag16L [35,38]. Relatively little is known about the function of either Pcdp1 or Spef2. The *Chlamydomonas reinhardtii* homolog of Pcdp1, FAP221, was shown to associate with a protein complex that localizes to the central pair apparatus of the cilium and regulates motility in a calcium-dependent pathway [47]. Spef2 was shown to associate with intraflagellar transport protein IFT20 in the testis [48], and its likely *C. reinhardtii* homolog also associates with the central pair apparatus [49]. Absence of laterality defects in both models is likely due to the localization and function of these proteins at the central pair apparatus, which is absent in the nodal cilia required for embryonic left-right patterning [41,42]. It is unknown whether Pcdp1 and Spef2 have related functions in the central pair apparatus, and the mechanisms by which these two proteins regulate mammalian ciliary function remains elusive.

Microscopic analysis of inflammatory cells in the airway and flow cytometric analysis of cytokine levels are consistent with an enhanced acute inflammatory response, particularly in the *bgh* mutant. The typical response to *S. pneumoniae* infection involves initial clearance by activated resident macrophages, followed by recruitment of neutrophils, monocytes, and lymphocytes from the bloodstream [2,4,43,50]. Kadioglu et al. showed that, in mice, the number of neutrophils and infiltrating macrophages increases within the first 12 to 24 hours after *S. pneumoniae* infection, with lymphocytes infiltrating by 48 hours post-infection [51]. Both the *nm1054* and the *bgh* mutant show a shift toward an activated macrophage and infiltrating lymphocytic response by the second day after infection. A milder response is seen in infected wild type animals, where the shift to macrophage activation is not evident until day 3, and the lymphocytic recruitment remains relatively low. By day 3, the *bgh* animals exhibit a dramatic lymphocytic response, with T lymphocytes comprising more than 80% of the cells isolated from the BAL fluid. A small number of neutrophils were observed in the BAL fluid from each mutant, but this was lower than 5% at all time points. While it is possible that the response in these PCD mutants is primarily macrophagic and lymphocytic, it is more likely that there is an early and brief neutrophilic response, after which the number of neutrophils drops dramatically and is replaced by lymphocytes and macrophages by day 2 post-infection.

The acute inflammatory response observed in BAL fluid after infection is supported by quantification of cytokine levels in the lung. There is a statistically significant increase in IL-6 by day 1 in the *bgh* mutant and by day 3 in the *nm1054* mutant compared to wild type. IL-6, which is secreted by resident macrophages and plays a role in both neutrophil recruitment and T cell differentiation, is up-regulated in multiple pulmonary inflammatory diseases and is a consistent marker of early inflammation [4,52,53]. In addition, mice lacking IL-6 have an impaired immune response to *S. pneumoniae* infection, with mutants displaying a higher lung bacterial titer, increased levels of pro-inflammatory and anti-inflammatory cytokines, and increased morbidity [54], further demonstrating that IL-6 plays an important role in early host defense. Therefore, the high levels of IL-6 in *nm1054* and *bgh* lungs indicate the onset of a more persistent inflammatory response in both PCD mutants than infected wild type animals. Increased secretion of TNF, which is secreted by macrophages and, along with IL-6, stimulates production of several other cytokines, is also associated with an early inflammatory response [55]. TNF levels following infection are only increased in the *bgh* mutant, further suggesting that the immune response is more severe in the *bgh* mutant than the *nm1054* mutant. The increase in TNF levels in the *bgh* lung is statistically significant on day 1 but loses significance thereafter, likely due to variability observed in TNF levels in the sampling of mutant mice.

IL-2 plays a critical role in driving the lymphocytic response [56]. Consistent with an increased number of lymphocytes in BAL fluid from both PCD mutants, there is a statistically significant increase in IL-2 levels in the *bgh* mutant by day 2 and in the *nm1054* mutant by day 3 relative to infected wild type animals. IL-2 is secreted by naïve T cells and promotes their differentiation into Th1 helper cells, Th2 helper cells, Th17 helper cells, and T regulatory cells, which in turn secrete IFNγ, IL-4, IL-17, and the anti-inflammatory cytokine IL-10, respectively [56,57]. A statistically significant increase in IFNγ, IL-4, IL-17, and IL-10 levels was observed in *bgh* mutants. The increase in IL-10 lost its statistical significance after day 1, but like TNF, this is likely due to variability in the sample population. In contrast, there is no statistically significant change in the levels of any of these T helper or T regulatory cytokines in the *nm1054* lung relative to the wild type, providing further evidence that the *bgh* mice have a more severe lymphocytic response to bacterial infection.

Increases in lymphocytic cytokines in the *bgh* lung are particularly important given their critical roles in the lymphocytic response. IL-17, which has a pro-inflammatory function in further recruitment of circulatory macrophages and neutrophils, is typically elevated in patients with a variety of pulmonary inflammatory diseases [58,59], and mice lacking the IL-17 receptor A show an enhanced susceptibility to *Klebsiella pneumoniae* infection and increased morbidity [60]. To prevent excessive damage to the lung, the anti-inflammatory cytokine IL-10, which is secreted by T regulatory cells, plays a critical role in mediating the lymphocytic response by inhibiting naïve T cells, Th1 cells, and Th2 cells [4,61-63]. This role was demonstrated in mice inoculated with both *S. pneumoniae* and IL-10, which resulted in a higher bacterial titer, decreased levels of pro-inflammatory cytokines, and increased morbidity [64], while infection with *Aspergillus fumigatus* in mice lacking IL-10 results in an enhanced inflammatory response and higher levels of pro-inflammatory cytokines in the airway [63]. There is an upward trend in IL-10 levels in *bgh* mice relative to wild type controls, and despite a lack of statistical significance, these increases are greater than those observed for the Th1, Th2, and Th17 cytokines. This finding suggests that there may be an activation of the anti-inflammatory response to mitigate damage to the lung. Further analyses are required to fully determine the extent of tissue damage in mutant lungs over the course of infection and the potential interventions that can be used to limit this damage in PCD patients.

## Conclusions

The findings in this study establish that mice lacking either Pcdp1 or Spef2 are distinct, clinically relevant models with an enhanced lymphocytic response to pulmonary infection with *S. pneumoniae*, a common respiratory pathogen in patients with PCD. This is particularly significant given that the pulmonary immune response to *S. pneumoniae* has not been previously investigated in murine models of PCD, and it strongly suggests that further research may be necessary with additional bacterial, viral, and environmental challenges. Future analyses of inflammatory response beyond the acute phase of infection may provide additional evidence of compromised pathogen clearance in these models. The effect of this compromise on pulmonary function, mortality, and susceptibility to infection also warrants further study. Differences in the severity of response to infection in the *nm1054* and *bgh* mutants may be due to differences in Pcdp1 and Spef2 protein function that could have differential effects on mucociliary clearance. Elucidating the biochemical mechanism by which these proteins regulate ciliary motility will ultimately uncover their roles in pathogen clearance and host defense.

## Abbreviations

BAL: Bronchoalveolar lavage; bgh: *big giant head*; CBF: Ciliary beat frequency; CCSP: Clara cell secretory protein; IFNγ: Interferon gamma; IL: Interleukin; PCD: Primary ciliary dyskinesia; Spag: Sperm associated antigen; Spef2: Sperm flagellar protein 2; TNF: Tumor necrosis factor.

## Competing interests

The authors declare that they have no competing interests.

## Authors’ contributions

CWM, JK, and TM performed the experiments and analyzed the data. GT, PV, and VCH assisted with experimental design and data analysis. LL designed the study, oversaw the research, and wrote the manuscript. All authors read and approved the final manuscript.

## Supplementary Material

Additional file 1 **Mean alveolar chord length in mutant lungs.** Alveolar chord lengths were measured in sections of wild type, *nm1054*, and *bgh* lungs. Lengths are not statistically different between mutant and wild type. Click here for file

Additional file 2 **Body weights after infection with *S. pneumoniae.*** Body weights were measured for wild type (A), *nm1054* (B), and *bgh* (C) mice prior to infection (Day 0) and on the day of euthanasia and analysis (Day 1, Day 2, or Day 3). To normalize the data, weight values are presented as percentage of initial weight (Day 0) on the day of analysis. Body weights are largely unaffected for all mice euthanized for analysis. Click here for file

Additional file 3 **Levels of *S. pneumoniae *** **in the mutant respiratory systems.** Bacterial counts were determined in the nose (A) and lung homogenates (B) of wild type, *nm1054*, and *bgh* animals on days 1, 2, and 3 after infection with *S. pneumoniae*. Counts are not statistically different between mutant and wild type. Click here for file
