# Supplementary figures and images for: Enhanced response to pulmonary Streptococcus pneumoniae infection is associated with primary ciliary dyskinesia in mice lacking Pcdp1 and Spef2
Source: Cilia. 2013 Dec 20;2:18. doi: 10.1186/2046-2530-2-18 (PMC3878133; doi:10.1186/2046-2530-2-18)

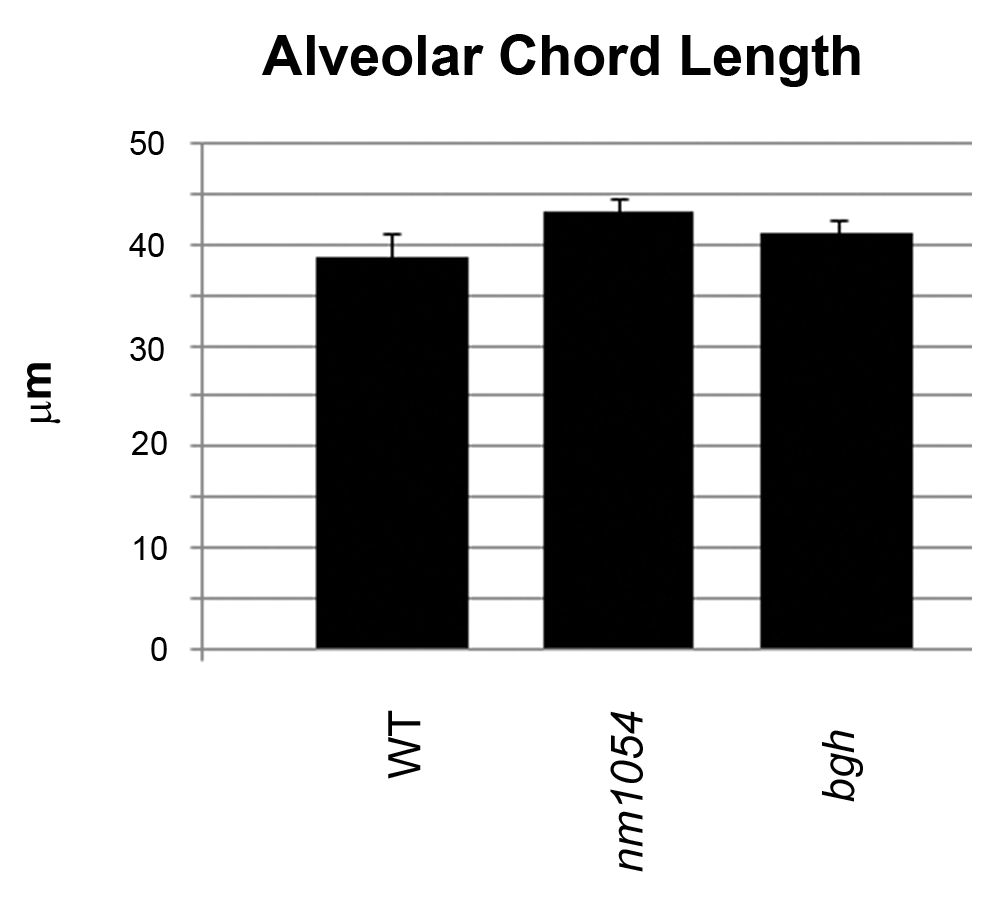

Supplement: Additional file 1 — Mean alveolar chord length in mutant lungs. Alveolar chord lengths were measured in sections of wild type, nm1054, and bgh lungs. Lengths are not statistically different between mutant and wild type. [file 2046-2530-2-18-S1.tiff]

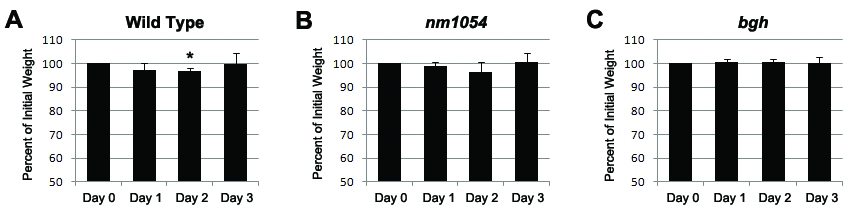

Supplement: Additional file 2 — Body weights after infection with S. pneumoniae. Body weights were measured for wild type (A), nm1054 (B), and bgh (C) mice prior to infection (Day 0) and on the day of euthanasia and analysis (Day 1, Day 2, or Day 3). To normalize the data, weight values are presented as percentage of initial weight (Day 0) on the day of analysis. Body weights are largely unaffected for all mice euthanized for analysis. [file 2046-2530-2-18-S2.tiff]

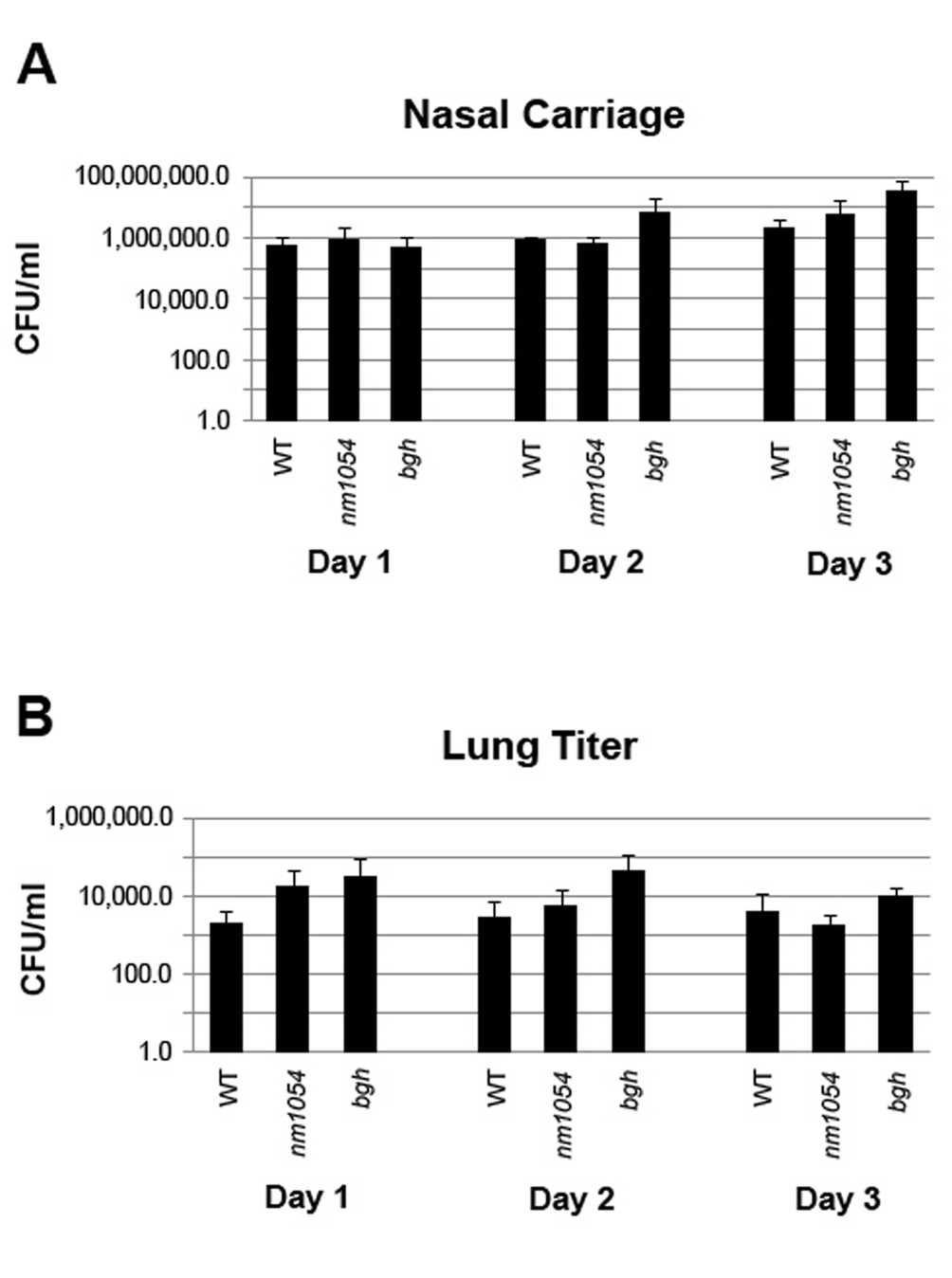

Supplement: Additional file 3 — Levels of S. pneumoniae in the mutant respiratory systems. Bacterial counts were determined in the nose (A) and lung homogenates (B) of wild type, nm1054, and bgh animals on days 1, 2, and 3 after infection with S. pneumoniae. Counts are not statistically different between mutant and wild type. [file 2046-2530-2-18-S3.tiff]
